# Stochasticity and the limits of molecular signaling in plant development

**DOI:** 10.3389/fpls.2022.999304

**Published:** 2022-10-19

**Authors:** Philip M. Lintilhac

**Affiliations:** Department of Plant Biology, The University of Vermont, Burlington, VT, United States

**Keywords:** stochasticity, stress-mechanics, organogenesis, surface shape, cell walls, turgor pressure, plant developmental information systems

## Abstract

Understanding plant development is in part a theoretical endeavor that can only succeed if it is based upon a correctly contrived axiomatic framework. Here I revisit some of the basic assumptions that frame our understanding of plant development and suggest that we consider an alternative informational ecosystem that more faithfully reflects the physical and architectural realities of plant tissue and organ growth. I discuss molecular signaling as a stochastic process and propose that the iterative and architectural nature of plant growth is more usefully represented by deterministic models based upon structural, surficial, and stress-mechanical information networks that come into play at the trans-cellular level.

## Introduction

As plant biologists we all learn to interpret the patterns of plant materials seen in section. This is only possible because plant cells cannot separate after cytokinesis, so the tissue patterns and cell-to-cell relationships established during division are retained and can be used to reconstruct the cell division activities and cell lineages of the growing organ. But animal cells are free to separate from each other and can migrate to new locations after division, obliterating any tissue patterning imposed by cell division itself. One consequence of this is that multicellular structural relationships and the physical signaling pathways they enable cannot be maintained as rigorously in animals as they are in plants. From a broader perspective, movement is fundamental to animal life. Animals need to move to find food, and evolution has provided them with tools suited to that end. Highly developed sensory systems, the ability to manage explosive muscle contraction, and the neurological integration that enables animals to interpret their environment in real time, all reflect complex developmental programs that are not found to the same extent in the plant kingdom.

But does this mean that developmental programming is simpler in the plant kingdom than it is in animals? Do the patterns that we see in the anatomy of multicellular plant organs and tissues reflect less highly evolved morphogenetic control systems? In this essay I try to formalize what many of us have intuitively suspected for many years: that developmental programming in the plant kingdom is in many respects more architecturally integrated and more spatially deterministic than the complex molecular control networks that evolution has made use of in the animal kingdom.

## The prevailing narrative

When we consider development in terms of the cellular processes upon which life is built, we tend to think in molecular terms. Molecular thinking brings cellularity into the tangible world of things that can be named, manipulated, and even synthesized. Molecules can be thought of as the citizens of a cellular society. They can recognize and exchange information with other molecules. They can archive information for the next generation. Molecules can be visualized. We can see their structures in our minds. We can attach markers to them and follow them around the cell and even edit the genetic templates that they derive from. So, it is not surprising that we think of developmental differentiation events, and their associated signaling pathways, as depending upon the exchange of molecular information and the migration of key molecular species from one part of a cell to another, or from one part of an organism to another. We interpret hormonal messaging, cellular signal cascades, and the induction of cellular differentiation in terms of a kind of molecular quorum-sensing; meaning that members of the correct molecular species must appear in sufficient number, at the right place, and at the right time to elicit a cellular response.

But molecular signaling, is fundamentally a stochastic process (Losick and Desplan, 2008), which is to say it involves an element of randomness (Bailey, 1964). Moving molecules is like herding cats. It does not necessarily lead to deterministic outcomes. So, it is reasonable to ask whether there are other kinds of information that can overcome some of the limitations of molecular signaling. Recent advances in plant cell and tissue biomechanics (Weise and ten Tusscher, 2019) are beginning to expose structural, mechanical, and surficial relationships that make up networks of geometrically precise and environmentally robust decision-making circuits uniquely adapted to the sessile, but architecturally sophisticated, growth-habit of the land plants. Physical signaling networks can be found wherever life has evolved, but in the plant kingdom it can be argued that the structural necessities of plant growth have promoted them as the primary choreographer of organogenesis.

## An evolutionary perspective

When plants first emerged onto dry land the constraints on growth and form were immediate and dramatic. Buoyant support vanished. Competition for sunlight made it necessary to raise a vertical axis above the land surface, which in turn required the transport of water to the growing tip. Gravitational loads meant that structural adaptations became paramount, driving the evolution of the cell wall, both at the ultrastructural level and at the level of the apoplast as a whole (Niklas and Spatz, 2012).

The evolution of the cellulosic cell wall also enabled plant cells to develop significant turgor pressures which could be used to drive the volumetric growth of cells and organs. In these high stress growth environments cell wall placements and orientations became critical. The release of growth forces into the surrounding tissues by local cell expansion could be used to transmit stress-mechanical information instantly, accurately, and with no dependence on molecular identity and stochastic molecular signaling. New kinds of control circuitry became possible, linking tissue architecture, organ topography, and morphogenetic behavior in deterministic feedback loops that support the iterative nature of plant growth (Lintilhac, 2014).

## The limits of molecular signaling

Molecular signaling in dissipative systems is difficult, particularly where the census number of any signaling molecule is low or when environmental noise is high, and although molecular signals can be highly selective in targeting specific receptors and eliciting specific responses, they require the transport of molecules from one location to another in roughly stoichiometric numbers, regardless of environmental noise. In the intracellular environment this kind of directed transport can be accomplished by enclosing populations of molecules in membrane-bound vesicles which can then be moved to specific locations within the cell, but molecule-specific trafficking mechanisms are not available when the target is beyond the reach of symplastic transport.

Physical signaling networks, on the other hand, are clearly deterministic. Force transmission is directional, instantaneous, and robust. It can effect action at a distance without molecular transport of any kind; and given the strong and permanent mechanical coupling between cells in the plant kingdom, and the ability of growing tissues to generate significant stress intensities, it seems reasonable to assume that evolution would have found ways to explore and recruit material behaviors and stress-mechanical relationships (Hernandez-Hermandez, 2014) to coordinate cellular proliferation, organic form, and the precise placement of new division walls (Facette et al., 2019).

Plant apical meristems are shape generators. But shape is more than just an outcome of tissue growth. The surface topography of a growing structure is itself a controlling variable. Free surfaces impose simple rules on the behavior of principal stresses (Heywood, 1969) acting as waveguides that channel force transmission to reshape the underlying stress fields (Frocht, 1962). Taken as a whole, these physical and material feedbacks are demonstrably critical during plant development. They integrate material behaviors from the nano-structural level to the level of whole tissues and organs, providing robust, instantaneous, and highly directional signals that can be interpreted at the cytoskeletal level and acted upon at the cell and tissue levels, resulting in the precise division wall orientations that we see everywhere in embryonic plant tissues (Jackson et al., 2018). We can begin to think of morphogenesis in the plant kingdom as an emergent manifestation of physical and surficial feedback circuits that regenerate themselves without direct genetic scripting (Lintilhac, 2014).

However, addressing the deficiencies in our understanding of plant development requires more than just an acknowledgement of the limits of stochastic molecular signaling. We need to be able to visualize and document the networks of physical interactions that control morphogenesis at the tissue and organ levels. Molecular signals can be visualized and followed in various ways, including the use of fluorescent probes; but there are no fluorescent probes for tension and compression. Transmitted force is essentially invisible, making it difficult to understand how it is interpreted at the cellular level. For instance, we need to understand the nature of the relationship between transmitted force and the positioning of the cell plate during cell division. How does the cell resolve and respond to the forces acting on it?

It has been known for many years that the patterns of division wall placements we see in actively dividing plant tissues reflect the principal stress fields radiating through appropriately configured photoelastic models (Lintilhac and Vesecky, 1984). But we need new experimental tools that will allow us to simplify stress-mechanical relationships and isolate critical variables. We need to develop experimental systems where single plant cells can be subjected to explicitly defined stress-mechanical inputs (Grasso and Lintilhac, 2016), and the resulting cellular behaviors can be more precisely monitored. Ultimately, we will need new and innovative ways to map the biomechanical landscape of organogenesis.

Historically, visualizing stress distributions and separating their tensile and compressive components was accomplished with photoelastic stress analysis (Lintilhac, 1974), a modeling technique which has largely been superseded by computer-based finite-element modeling. More recently however, photoelastic analysis has re-emerged in the form of Digital Photoelastic Analysis (Solaguren-Beascoa, 2009), which combines the visual immediacy that derives from photoelastic rendering with the convenience of digital extraction of principal stress trajectories. Now we need to look more deeply into the physical and biomechanical circuitry that coordinates plant development in a robust, deterministic, progression of morphogenetic changes.

## The trans-cellular domain

In order to clarify the distinctions between various intracellular signaling processes and their extracellular and tissue-level counterparts I am introducing the concept of the *trans-cellular* domain as a regime of multicellular informational channels that extends beyond the intracellular cytoplasmic domain, beyond the symplast, and beyond the apoplast. The trans-cellular domain comprises whole-cell multicellular information channels that span multiple individual cell lengths, and which are connected both symplastically and apoplastically to create a variety of integrated networks over which physical and structural information can be transmitted at the tissue and organ levels. Most physical signals acting through growing plant tissues would be considered to propagate in the trans-cellular domain because they are necessarily reflected in osmotic and metabolic changes at the level of the single cell, and structural, ultrastructural, and biomechanical changes at the apoplastic level.

## Conclusion

One of the most vexing problems confronting researchers and students of plant development and morphogenesis is the difficulty in reconciling stochastic hormonal and biochemical signaling systems with the structural precision and architectural fitness of cell division and cell wall installation in growing plant tissues. In many instances molecular information transfer appears to break down because of its inherent spatial imprecision and its sensitivity to environmental noise. But by taking advantage of the mechanical continuity of plant tissues, molecular signals that originate in stochastic cellular processes can be translated into deterministic physical signals that are precise, instantaneous, and robust in the face of environmental noise. Cell and tissue mechanics, rather than being simply an interesting sub-discipline of plant developmental biology, become the language through which critical developmental information is transmitted at the trans-cellular level of whole tissues and organs. Interpreting the choreography of plant development in terms of deterministic, biophysically integrated behaviors operating at the trans-cellular level complements our understanding of transcription-directed signaling in land plant development, but it also offers the prospect of being able to trace many aspects of plant development and morphogenesis back to physical first principles and axioms.

## Data availability statement

The original contributions presented in the study are included in the article. Further inquiries can be directed to the corresponding author.

## Author contributions

The author confirms being the sole contributor of this work and has approved it for publication.

## Funding

This work was supported by SPARK-VT seed funding awarded by the University of Vermont's Office of the Vice President for Research.

## Conflict of interest

The author declares that the research was conducted in the absence of any commercial or financial relationships that could be construed as a potential conflict of interest.

## Publisher’s note

All claims expressed in this article are solely those of the authors and do not necessarily represent those of their affiliated organizations, or those of the publisher, the editors and the reviewers. Any product that may be evaluated in this article, or claim that may be made by its manufacturer, is not guaranteed or endorsed by the publisher.
